# Depressive Symptoms Among Older Gay Men: What Are the Most Important Determinants?

**DOI:** 10.3390/healthcare13030216

**Published:** 2025-01-21

**Authors:** Hala Asmer Khoury, Tova Band-Winterstein, Yaacov G. Bachner

**Affiliations:** 1 Department of Gerontology, University of Haifa, Haifa 3498838, Israel; hala.asmer@gmail.com (H.A.K.); twinters@research.haifa.ac.il (T.B.-W.); 2 Minerva Center on Intersectionality in Aging, University of Haifa, Haifa 3498838, Israel; 3 Department of Epidemiology, Biostatistics and Community Health Sciences, Faculty of Health Sciences, Ben-Gurion University of the Negev, P.O. Box 653, Beer Sheva 8410501, Israel

**Keywords:** depression, older, gays, loneliness, internalized homophobia, self-esteem, ageism, health behavior

## Abstract

**Background:** Studies have shown that gay men experience higher levels of depression and are more likely to report suicidal ideation, plans, and attempts over their lifetime compared to heterosexual men. However, most studies have been conducted with adolescents and young adults, while there is a lack of research focusing on older adults. The aims of this study are to assess the level of depressive symptoms among older gay men and examine the associations between five key factors—loneliness, internalized homophobia, self-esteem, ageism, health behavior—and depressive symptoms. **Methods:** The convenience sample included seventy-nine gay men living in the community. Prospective participants were recruited by facilitators of social and support groups, who either distributed the questionnaire directly to members on-site or forwarded a link to their emails. All study measures used were valid and reliable. **Results:** Participants’ mean level of depression exceeded the scale’s cutoff point for detecting depression, indicating mild depression. Four variables made a significant contribution to the explanation of depression, with loneliness having the largest contribution, followed by ageism, internalized homophobia, and health behavior. The regression model explained a very high percentage of the depression variance (83%). **Conclusions:** These four factors are central to understanding depression among older gays. Medical and social professionals should recognize their significance and incorporate them into the treatment provided to those in need. Further studies are needed to gain a deeper understanding of the factors associated with depression in this vulnerable population.

## 1. Introduction

Same-sex relations are common across different cultures and were reported as early as ancient Greece [1]. The percentage of gay men in studies across different countries ranges from 6% to 10%. This variance is likely due to the way surveys are structured and administered. Studies conducted among the Jewish population in Israel, aged 18 to 45, found that 8.2% of men identified themselves as gay or bisexual, 10.2% reported sexual experiences with members of the same sex, and 11.3% revealed that they are attracted to individuals of the same sex [2,3]. No estimates exist for older age groups. However, based on estimates of the share of LGBT adults in the population, we estimate that there are between 36,920 and 110,760 LGBT adults aged 55 and older in Israel [4].

The process of developing a same-sex identity is unique and involves characteristics distinct from those in the sexual identity development process of the general population. It encompasses complex components such as coping with social pressure, behaviors, and personality traits related to sexual orientation and dealing with social rejection associated with this identity [5]. This uniqueness, along with its complex implications, forms the basis for considering individuals with same-sex identity as a minority group.

Belonging to a minority group is usually associated with various stresses. These stressors are different from other life stressors because they add to the pressures faced by society as a whole. This type of stress is chronic and stable, rooted in social and cultural components, and arises as a result of them. This means that such stressors are caused by societal norms, which arise from social processes, institutions, and structures, rather than from events or situations directly related to the individual.

Developed by Meyer [6], the minority stress model emphasizes the significant role of stressors uniquely faced by members of minority groups, including violence, stigma, and discrimination directed at them, which can lead to negative physical and mental health outcomes [7]. According to this model, minorities often encounter both group-specific distal and proximal stressors that combine to create conditions of heightened stress [6,8]. Distal stressors refer to external events, such as interpersonal victimization and structural discrimination, while proximal stressors encompass the internal struggles minority group members experience in response to these external pressures [7,8,9]. In the literature on minority stress, depression, suicidal ideation, and behavior have increasingly been recognized as critical indicators of poor mental health among individuals facing these specific stressors [9,10]. The minority stress model has been a guiding framework in research on the mental health and well-being of sexual minorities and serves as the theoretical foundation for this study.

Years of research have shown that negative mental health outcomes (e.g., depression) among individuals from sexual minorities are attributed to a unique set of stressors, including violence, stigma, and discrimination [6,8]. In addition to these stressors, older individuals within sexual minorities face a distinct set of challenges associated with aging, such as changes in health, the narrowing of social networks, and ageism [11]. Therefore, older adults from sexual minorities face double marginality and dual challenges of coping with both their sexual orientation and the aging process.

### 1.1. Depression Among Older Gay Men

Studies have shown that gay men experience higher levels of depression and anxiety disorders and are more likely to report suicidal ideation, plans, and attempts over their lifetime compared to heterosexual men [12,13]. For instance, a study conducted in the United States found that gay men are 3.57 times more likely to experience depressive disorders and 5.09 times more likely to experience panic disorders compared to heterosexual men [14]. Additionally, one in six gay men has attempted suicide at some point in their life [15]. While much of the research has focused on sexual minority youth, there has been less attention given to elderly sexual minorities, despite the aging of the population. Evidence from a meta-analysis of 12 UK population health surveys indicates that sexual minority older adults (aged 55 or older) have 2.24 times higher odds of experiencing depression compared to heterosexuals of a similar age, whereas sexual minority youth (under the age of 35) have 1.92 times higher odds of depression compared to their heterosexual peers [16]. Moreover, a population health survey of adults aged 50 and above in Washington State found that lesbian, gay, and bisexual individuals reported poorer mental health compared to their heterosexual peers [17]. In a follow-up study in 2017, these researchers observed increased odds of psychological distress among these individuals [18]. Another study, drawing data from the Canadian Longitudinal Study on Aging (adults aged 45–85), found a significant effect of sexual orientation on depressive symptoms, with gay identity being significantly associated with symptoms of depression [19]. In another study of older gay men with a mean age of 70 from Spain and Portugal, depressive symptom levels were found to be above the normal cutoff point on the scale [20]. Depression in older adults has been associated with a higher risk of mortality, disability, and suicidal thoughts and attempts, as well as being a contributing factor to cognitive decline and dementia [21].

Hence, based on a comprehensive literature review, this study examines among older gay men factors derived from the minority stress model and its related aspects (e.g., internalized homophobia, self-esteem, health behavior), as well as those more closely linked to the aging process (e.g., loneliness, ageism).

### 1.2. Correlates of Depression Symptomatology

Loneliness: Loneliness is a subjective feeling defined as the gap perceived by an individual between the number of social connections they desire and the number they actually have [22]. While loneliness is common among the elderly, it is even more prevalent among individuals from sexual minorities. For example, a Dutch study reports that lesbian women, gay men, and bisexuals aged 55–89 are approximately 1.5 to 2 times more likely, respectively, to experience loneliness compared to their heterosexual counterparts of the same age [23]. Loneliness is recognized as a significant source of stress, associated with a poor quality of life, and can lead to serious mental health issues, primarily depression and suicidal attempts. Older individuals from sexual minorities are more vulnerable to the negative effects of loneliness compared to their heterosexual peers, as many do not have children, are estranged from their families, and live alone. Those who live with a partner often do so secretly and lack legal recognition of their relationship. Living alone often evokes many times feelings of loneliness and depression due to the absence of a partner or family members with whom they can share emotional and instrumental support. Feelings of helplessness, rejection, and non-disclosure of sexual orientation are associated with lower social engagement within the broader community, resulting in subjective feelings of loneliness [24]. Moreover, concerns about revealing their true selves can lead to a lack of meaningful connections and feelings of social invisibility among family and peers. In a recent prospective cohort study conducted in England, after adjustments, high scores of depressive symptoms and loneliness were found among sexual minorities, along with a positive association between the two. Mediation analyses showed that loneliness explained 15% of the association between sexual orientation and subsequent depressive symptoms [25]. Similarly, a recent longitudinal study found an association between loneliness and depression in older sexual minority men, both with and without HIV [26].

Internalized homophobia: Internalized homophobia (also referred to as internalized homonegativity and internalized heterosexism) is a common source of minority stress, defined as the process by which lesbian, gay, and bisexual individuals internalize and direct societal homophobic attitudes toward themselves [6]. It results from living in a heteronormative society, and its dimensions include negative overall beliefs about same-sex attraction, discomfort with revealing one’s sexual orientation, a sense of detachment from other gay and bisexual individuals, and discomfort with engaging in same-sexual activity [27]. Internalized homophobia has been identified in many studies as a significant risk factor for poor mental health, especially depression and suicidal ideation. For example, a recent study found that older gay and bisexual men with higher levels of sexual stigma presented significantly higher anxiety, depression, somatization, and suicidal ideation scores [28]. In another meta-analytic study involving 31 studies, a small to moderate effect size was found between internalized homophobia and mental health, with a stronger association observed with depression compared to anxiety. This effect was more pronounced in samples with a higher mean age of participants [29]. It is important to note that age-related differences in internalized homophobia have been observed, with older gay adults showing a higher prevalence of internalized homophobia compared to younger adults. Furthermore, individuals with high levels of internalized homophobia were found to be at a significantly greater risk for depressive symptoms than those with lower levels [30].

Self-esteem: High self-esteem involves self-respect and a sense of personal worth [31]. Studies indicate that individuals belonging to sexual minority groups report lower self-esteem compared to heterosexual cisgender groups [32,33]. The internalization of negative attitudes and stigma can adversely affect the self-esteem of gay men, potentially leading to maladaptive behaviors and emotional disturbances, such as depression, anxiety, substance use, and risky sexual behavior [34]. A study among older lesbian, gay, and bisexual adults found that higher self-esteem was correlated with better mental health and fewer lifetime suicidal ideation [35]. In a systematic review of the literature on psychosocial factors and aging in older lesbian, gay, and bisexual individuals, it was found that, overall, higher self-esteem was significantly correlated with better mental health [36]. A review of the literature indicates a lack of studies directly examining the relationship between self-esteem and depression, with most existing research primarily focusing on young gay adults. One of the aims of the current study is to explore this association among older gay men.

Health behavior: Health behavior is defined as behavior that directly or indirectly impacts an individual’s health [37]. The literature distinguishes between health-promoting behaviors and health-risk behaviors. The latter include daily lifestyle patterns such as alcohol and drug use, behaviors that contribute to injuries and violence (including suicide), smoking, unhealthy dietary habits, and sexual behavior [16]. Studies report that sexual minorities are more likely to smoke and drink than their heterosexual peers [16,38,39]. However, research indicates that sexual minorities tend to engage more in physical activity during adulthood compared to their heterosexual counterparts [38]. A study conducted among same-sex male couples observed a high prevalence of drug use and binge drinking. It also found an association between self-reported clinically significant depressive symptoms and an increased likelihood of polydrug use [40]. Another study found that men with HIV and higher levels of depression were more likely to report being drunk in the past three months compared to those with lower levels of depression. Additionally, higher levels of depression were associated with increased drug use during this period [36].

Ageism: Ageism is defined as discrimination based on age arising from the prejudice of one age group against another [41]. Similar to self-stigma, older adults often internalize the negative aspects of aging and reinforce the stereotypes directed at them. Allen [42] states that ageism becomes internalized when older individuals adopt negative societal stereotypes about aging as part of their self-identity. This internalization acts as a form of stress that may lead to negative physical and mental health outcomes [42,43]. A study conducted with American gay men (average age 61) found a positive correlation between internalized gay ageism and depressive symptoms, with internalized gay ageism accounting for 18% of the variance in depressive symptoms [44]. Another study comparing middle-aged and older Israeli gay men to their heterosexual peers found that the association between negative attitudes toward aging and depressive symptoms was moderated by sexual orientation. Specifically, negative attitudes toward aging were more strongly associated with depression among gay men than among heterosexual men [45]. Additionally, a qualitative study conducted in Israel among gay men and lesbians identified negative attitudes toward aging as a central theme, which manifested as ageism [46].

The aims of the study are twofold:

1. To assess the level of depressive symptoms among older gay men.
2. To examine the associations between loneliness, internalized homophobia, self-esteem, ageism, health behaviors, and depressive symptoms.

A deeper, more comprehensive understanding of the factors contributing to depression will help develop targeted interventions designed to support this vulnerable community.

## 2. Materials and Methods

### 2.1. Participants and Procedure

Seventy-nine older gay men participated in the study. The inclusion criteria were 55 years of age or older, self-identification as gay, and the ability to read and understand the study questionnaire. Prospective participants with active mental illness and those who used psychiatric medicines were excluded. The age of 55 was determined because literature reports that gay men experience accelerated aging processes due to stressors related to their gender and sexual identity and sometimes also long-term exposure to stress hormones [47].

We distributed the study questionnaire digitally using QUALTRICS XM software. Prospective participants accessed the questionnaire via an internet link sent by email. Potential participants were reached through contacts and facilitators of gay groups for individuals aged 55 and over, who then forwarded the questionnaire to their email distribution lists. Additionally, we attended support groups for older gay men and distributed the questionnaire directly to the members.

The research questionnaire was anonymous, ensuring that participants could not be identified. At the beginning of the questionnaire, the purpose of the study was explained, along with a statement emphasizing that participation was entirely voluntary. Participants were also informed that they could withdraw at any time and that the data collected would be used solely for the specific purposes of the study. Entering the online questionnaire constituted informed consent to participate in the study. The study was approved by the Ethics Committee of the University of Haifa.

### 2.2. Instruments

Depression: Measured by the Center for Epidemiological Studies (CES-D) depression scale [48] designed to assess depressive symptoms in the general population. The scale includes 20 items rated on a four-point Likert-type scale where 0 = rarely or never, 1 = some or a little of the time, 2 = sometimes or some of the time, 3 = most of the time or all of the time. An overall score was created by summing the responses to all items so that a higher score represents a higher sense of depression. The internal consistency was high (α = 0.95).

Loneliness: Measured using the R-UCLA scale [49]. In the present study, the abridged version (8-ULS) was used, which includes eight items rated on a 4-point Likert-type scale ranging from 1 = never to 4 = often. An overall score was created by summing the responses to all items so that a higher score represents a stronger sense of loneliness. The internal consistency was high (α = 0.91).

Internalized Homophobia: Measured using the Internalized Homonegativity Inventory (IHNI) scale [50]. This scale consists of 22 items rated on a 6-point Likert-type scale ranging from 1—“do not agree at all” to 6—“strongly agree”. The items are based on three factors—negative self-esteem, statements about same-sex attraction and their approval, as well as the ethics of same-sex identity. The items related to the self-esteem factor were omitted from the scale, as they were measured separately as an independent index. An overall score was created by averaging the responses to all items so that a higher score represents a stronger sense of internalized homophobia. The internal consistency was high (α = 0.92).

Self-esteem: Measured using the Rosenberg RSES self-esteem scale [31]. This scale contains 10 items rated on a 5-point Likert-type scale ranging from 1—“strongly disagree” to 5—“strongly agree”. An overall score was created by averaging the responses to all items so that a higher score represents a stronger sense of self-esteem. The internal consistency was high (α = 0.93).

Health behavior: This index was composed of four items: (1) Do you smoke? (2) Do you consume five or more alcoholic beverages on one occasion? [51] (3) Do you engage in physical activities such as gardening, housework, using stairs, sports, or any other activity that causes an acceleration of the heart rate and an increase in breathing? [52] (4) Do you suffer from sleep problems? All items were rated on a dichotomous scale (yes/no). The total index was created by summing the number of positive health behaviors. For example, a participant who does not smoke received one point; a participant who does not smoke and does not consume alcohol received two points; a participant who does not smoke, does not consume alcohol, and performs physical activity received three points; and a participant who does not smoke, does not consume alcohol, performs physical activity, and does not suffer from sleep problems received four points. It is important to note that although sleep problems are not considered a pure health behavior, they are strongly related to both physical and mental health and are known to be common among gay men, as documented in many studies [53,54]. Therefore, we included them alongside other health behaviors.

Ageism—Measured using the Index of Attitudes towards the Elderly [55]. The questionnaire includes 34 items, 17 positive and 17 negative, rated on a 6-point Likert-type scale ranging from 1—“strongly disagree” to 6—“strongly agree”. The positive and negative statements are presented in random order. To calculate the positive score, the positive items are summed separately from the negative items. A higher score on the positive scale indicates more favorable attitudes toward older adults, while the negative items are reverse-scored, with lower scores reflecting more unfavorable attitudes toward older adults. The internal consistency was acceptable (α = 0.81).

Socio-demographic characteristics: Details were collected regarding age (years), level of religiosity (secular, conservative, orthodox, ultra-orthodox), family status (married or living with a partner, other), cohabitation (alone or family/partner/friends), children (yes, no), education (years), economic status (ranging from 1—low economic status to 6—high economic status).

## 3. Data Analysis

The data were described using descriptive statistics (means, standard deviations, ranges). Associations between variables were analyzed using Pearson, Spearman, or chi-square tests according to scale structures. The unique contribution of the independent variables to the explanation of depression was assessed using multivariable linear regression. Only variables found to be significantly associated with depression in the bivariate analyses were included in the regression model. The internal reliability of the measures was calculated using Cronbach’s alpha. A significance level of *p* < 0.05 was set for all analyses. Data analysis was performed using SPSS software, version 28.

## 4. Results

The socio-demographic characteristics of the seventy-nine participants are described in Table 1. The mean age was 64 years (SD = 6.9), with a range of 55 to 79 years. Most participants were either not married or did not live with a partner and identified as secular. Three-quarters lived alone, and slightly more than half had children. The participants’ mean years of education were 16.1 (SD = 3.80), and they perceived their economic status as relatively good.

Descriptive statistics of the study measures and their correlation with depression are presented in Table 2. The distribution of depression level was normal (skewness = 0.26, kurtosis = −1.05). Participants’ perceived mean level of depression was notably high (M = 20.98, SD = 13.31) relative to the scale’s possible range (0–60), surpassing the cutoff point of 16 for detecting depression [48] (indicating mild depression). Furthermore, 47 out of the 79 participants (59.5%) scored above this threshold.

Significant positive and strong correlations were found between levels of loneliness, internalized homophobia, ageism, and depression. Significant negative correlations were observed between self-esteem, health behavior, and depression. This means that the higher the participants’ levels of loneliness, internalized homophobia, ageism, and lower self-esteem and health behavior, the higher their depression level.

A correlation matrix of all the independent variables associated with depression was conducted to examine multicollinearity. As shown in Table 3, the correlations among the different variables ranged from r = 0.10 to r = −0.72. These values were below the threshold of r = 0.80, which is commonly used to identify multicollinearity [56].

The independent variables found to be significantly associated with depression were examined as possible predictors of depression levels in a multiple linear regression analysis (Table 4). The regression model was significant {F(5, 79) = 74.4, *p* < 0.001}, with all variables explaining a high percentage of the variance in depression (R^2^ = 0.83; adj R^2^ = 0.82). Four variables were found to be significant predictors of depression. Loneliness made the largest contribution to the explained variance (β = 0.38, *p* < 0.001), followed by ageism (β = 0.28, *p* < 0.001), internalized homophobia (β = 0.22, *p* < 0.01), and health behavior (β = −0.11, *p* < 0.05). Self-esteem did not reach the required level for statistical significance, although it approached it (*p* = 0.06).

## 5. Discussion

As a sexual minority group, gay men are exposed to unique stressors related to their sexual identity, including violence, stigma, humiliation, and discrimination. These stressors, when combined with everyday life challenges, adversely affect their mental health, resulting in a higher prevalence of depression and suicidal tendencies [6,7,57]. Within this group, older gay men face additional challenges related to the aging process, compounding the stressors associated with their sexual orientation. The current study assessed the level of depressive symptoms among older gay men in Israel and examined the associations between five key factors—loneliness, internalized homophobia, self-esteem, ageism, and health behavior—and depressive symptoms.

The results of our study revealed a mild mean level of depressive symptoms among participants (M = 20.98, SD = 13.31), with scores above the scale’s cutoff point of 16 for detecting depression [48]. Notably, 47 out of 79 participants (59.5%) had depression scores exceeding the cutoff. This finding is consistent with previous research demonstrating elevated levels of depression among older gay men [6,16,19]. This finding should also be interpreted within the specific social and cultural norms prevalent in Israeli society. Israel is a multicultural society composed of various religious (Jewish, Muslim, Christian) and ethnic groups. Israel is known as a familistic and pronatalist state, where childbirth and parenthood are upheld as central values [58]. Despite general support for families, older gay men are less likely to be in relationships or have children, possibly due to gay men being specifically targeted by discriminatory practices in adoption or surrogacy, which limits their access to fatherhood, while women can receive state support for fertility treatments [59]. Gay men also often face greater discrimination due to widespread patriarchal and masculine norms in a more traditional society [60]. Additionally, Israeli gay men, particularly Jewish religious ones, often experience difficult feelings related to their Jewish identity and religion (as Judaism prohibits same-sex relations). The conflict between Judaism and gay identity can evoke painful experiences of internal contradiction, guilt, and depression [61]. Hence, Israeli society is generally characterized by social norms and religious traditions that disapprove of same-sex orientation alongside formal and hierarchical family dynamics [62]. These cultural norms and religious contexts probably intensify the stress experienced by individuals within sexual minorities. It is also important to note that the participants in our sample grew up during a time when same-sex relations were considered a criminal offense. It was only in 1988 that the Israeli parliament (Knesset) repealed this law, thereby decriminalizing same-sex relations [45,46,47,48,49,50,51,52,53,54,55,56,57,58,59,60,61,62,63].

The regression model explained a substantial percentage of the variance in depression (83%), highlighting the importance of the independent variables studied. Four out of the five factors emerged as significant predictors, with loneliness having the strongest contribution, followed by ageism, internalized homophobia, and health behaviors.

Unsurprisingly, loneliness emerged as the strongest predictor in our study, demonstrating a strong positive correlation with depression. While loneliness is widespread among older adults, it is especially prevalent among older gay men and has been reported to be significantly higher compared to heterosexual control groups [64,65]. Many gay men do not have children, live alone, and often conceal their romantic relationships. Additionally, fears and anxieties about revealing their sexual orientation can lead to social isolation, making it harder to form new friendships and diminishing the social support network available to them. This finding, consistent with prior research, highlights loneliness as a major factor associated with depression among older sexual minority populations [25,26,64,65].

Ageism emerged as the second strongest predictor, displaying a positive correlation with depression. This suggests that the more ageist attitudes participants held, the higher their levels of depressive symptoms. While most research tends to focus on the external experiences of age-related discrimination faced by older adults, far fewer studies examine the impact of individuals’ own internalized prejudices toward aging (i.e., internalized ageism). Studies have found that aging gay men tend to perceive the aging process more negatively and exhibit stronger ageist attitudes compared to their heterosexual counterparts [66,67]. This tendency is likely driven by the high value placed on physical appearance, youth, and sexual vitality within gay communities, which is reflected in their greater tendency to engage in physical activity during adulthood compared to their heterosexual counterparts [38]. These attributes often play a significant role in shaping one’s sense of self-worth [68]. Hence, older gay men may adopt the negative views toward aging prevalent in their community, which can lead to feelings of rejection and emotional distress [69]. Our finding is consistent with previous research, both Israeli and international, which reports a positive association between ageism among gay men and depressive symptoms [44,45].

Internalized homophobia emerged as the third strongest predictor, showing a positive association with depression. This finding is consistent with previous studies that have identified internalized homophobia as significantly correlated with impaired mental health, particularly depression and suicidal ideation [70]. According to minority stress theory [6], internalized homophobia is a significant source of stress. Belonging to a socially stigmatized group can lead to social isolation and marginalization, causing some men to feel unworthy and internalize the negative perceptions directed at them, which in turn are associated with depression. A factor that may strengthen the explanation for our finding is that older gay men have been found to exhibit a higher prevalence of internalized homophobia compared to younger adults. Furthermore, individuals with high levels of internalized homophobia have been found to be at significantly greater risk for depression than those with lower levels [30].

Finally, health behavior emerged as the weakest predictor of depression, as indicated by the lowest standardized beta value, in a negative direction. This means that better health behavior was associated with lower levels of depression. This finding aligns with the findings of many other studies that report an association between risky health behaviors and higher rates of depression among gay men [16,38,39,40]. It seems that one way to cope with minority stress and its emotional consequences is to engage in risky behaviors such as drinking, smoking, using illegal drugs, and practicing unsafe sexual intercourse. This may be especially true for older gay men who grew up in a time when the environment, including their families, was non-accepting, and same-sex relationships were considered deviant, immoral, and criminal.

As expected, self-esteem demonstrated a negative association with depression but was found not to be significant. However, its significance level approached statistical significance (*p* = 0.06). We believe that with a larger sample size, this variable would likely have been found significant.

## 6. Limitations of the Study

This study has several limitations that should be acknowledged. First, all participants were Jewish, while Israeli society also includes Arabs (both Muslims and Christians) and other minorities. Additionally, we did not inquire about the participants’ ethnic identities. Second, the sample size was small and based on convenience, non-random sampling. These factors limit the generalizability of the findings and their representation of the broader population of older gay men in Israel. Moreover, in small samples, there may be heterogeneity in responses, which could introduce biases. Furthermore, due to the small sample size, we were unable to adjust for the socio-demographic characteristics in the multivariate regression model. Third, the research questionnaire included sensitive personal and social variables, such as loneliness, internalized homophobia, self-esteem, and health behavior. In such cases, participants may have been influenced by a desire for social approval, potentially providing responses that conformed to socially expected norms. Despite these limitations, the findings of the study align with existing global literature and offer a unique perspective on the older gay men population in Israel.

## 7. Conclusions and Implications

This study contributes to the current knowledge of the significant determinants of depressive symptomatology among older gay men. Older Israeli gay men were found to experience mild depressive symptoms. This finding, consistent with many previous studies, further underscores the mental health vulnerability of older gay men. Loneliness, internalized homophobia, ageism, and health behavior are the primary factors contributing to the depressive experience.

Our findings have both theoretical and practical implications. On a theoretical level, the ability of these four factors to significantly explain depression underscores their importance and contributes to a deeper understanding of this experience. Therefore, future studies should further examine these factors with larger sample sizes, diverse populations, and longitudinal study designs. Special attention should be given to loneliness, which emerged as the most important factor in explaining depression.

On a practical level, medical and social professionals should recognize the importance of these factors and incorporate them into the treatment provided to this vulnerable population. Moreover, these factors should be considered in any intervention programs aimed at reducing depression among older gay men.

## Figures and Tables

**Table 1:** Participants’ socio-demographic characteristics (n = 79).

Variable	N (%)	Mean	SD
Age (years)		64.03	6.90
Range: 55–79			
Family status			
Married/with a partner	14 (17.7)		
Other	65 (82.3)		
Cohabitation			
Alone	59 (74.7)		
Family/partner/friend	20 (25.3)		
Children			
Yes	50 (63.3)		
No	29 (36.7)		
Education (years)		16.11	3.80
Range: 8–32			
Religiosity level			
Secular	59 (75.6)		
Conservative	19 (24.4)		
Perceived economic status		3.54	1.03
Range: 1–6 ^			

^ 1 = low, 6 = high.

**Table 2:** Descriptive statistics of study measures and their correlation with depression (n = 79).

Variable	Number of Items	Possible Range	Mean	Standard Deviation	Correlation with Depression
Depression	20	0–60	20.98	13.31	--
Loneliness	8	8–32	17.93	5.48	r = 0.84 ***
Internalized homophobia	22	1–6	2.28	0.90	r = 0.74 ***
Self-esteem	10	1–5	3.97	0.94	r = −0.71 ***
Ageism	34	1–6	3.29	1.33	r = 0.76 ***
Health behavior	4	0–3	1.19	0.80	r = −0.31 **

** *p* < 0.01, *** *p* < 0.001.

**Table 3:** Correlation matrix of independent variables (n = 79).

Variable	Loneliness	Internalized Homophobia	Self- Esteem	Ageism	Healthy Behavior
Loneliness	1	0.63 **	−0.69 **	−0.68 **	0.21
Internalized homophobia		1	−0.72 **	−0.57 **	0.10
Self-esteem			1	0.41 **	−0.12
Ageism				1	−0.25 *
Healthy behavior					1

* *p* < 0.05, ** *p* < 0.01.

**Table 4:** Predictors of participants’ level of depression—results of a multiple linear regression analysis (n = 79).

Predictor	B	S.E	β	t
Loneliness	0.91	0.20	0.38	4.47 ***
Internalized homophobia	3.18	1.14	0.22	2.78 **
Self-esteem	−2.20	1.15	−0.16	−1.90 ^
Ageism	2.83	0.71	0.28	3.94 ***
Health behavior	−1.86	0.82	−0.11	−2.26 *

R^2^ = 0.83; adj R^2^ = 0.82, ^ *p* = 0.06, * *p* < 0.05, ** *p* < 0.01, *** *p* < 0.001.

## Data Availability

Data regarding this study can be obtained from the authors upon request.
